# Quantum Games with Unawareness with Duopoly Problems in View

**DOI:** 10.3390/e21111097

**Published:** 2019-11-10

**Authors:** Piotr Frąckiewicz, Jakub Bilski

**Affiliations:** Institute of Exact and Technical Sciences, Pomeranian University, 76-200 Slupsk, Poland; jakub.bilski@apsl.edu.pl

**Keywords:** quantum duopoly, quantum game, game with unawareness

## Abstract

Playing the Cournot duopoly in the quantum domain can lead to the optimal strategy profile in the case of maximally correlated actions of the players. However, that result can be obtained if the fact that the players play the quantum game is common knowledge among the players. Our purpose is to determine reasonable game outcomes when players’ perceptions about what game is actually played are limited. To this end, we consider a collection consisting of the classical and quantum games that specifies how each player views the game and how each player views the other players’ perceptions of the game. We show that a slight change in how the players perceive the game may considerably affect the result of the game and, in the case of maximally correlated strategies, may vary from the inefficient Nash equilibrium outcome in the classical Cournot duopoly to the Pareto optimal outcome. We complete our work by investigating in the same way the Bertrand duopoly model.

## 1. Introduction

Quantum game theory [1] unites game theory with quantum mechanics. It is an interdisciplinary research field that assumes games to be played with the use of objects that behave according to the postulates of quantum mechanics. So far, there have been studied refinements of Nash equilibria in quantum games (e.g., evolutionarily stable strategies [2,3,4,5] or extensive-form games [6,7,8], correlated equilibria [9,10,11], repeated games [12,13], and also problems concerning cooperative games theory [14,15]. New ideas are still proposed. Studying quantum games with limited perception (with unawareness) [16,17] is one of the latest trends. Limited perception in games enables us to describe situations in which a player has his own view about the game and views how other players are considering the game. Regarding quantum games, the notion of unawareness provides us with the tools to consider problems in which some of the players perceive quantum games, whereas the other players may think they play the classical game.

The aim of this paper is to bring together the notions of game with unawareness and the notion of quantum duopoly. We shall introduce an element of unawareness to quantum versions of Cournot and Bertrand duopoly already studied by us in papers [18,19]. In particular, we shall consider cases in which players play the quantum duopoly game; however, some of the players may not realize that fact or the players may be aware of playing the quantum game, but at the same time may find that the other player views the classical game. Our investigation also covers higher-order iteration of awareness of the players, for example, one of the players considers the quantum game, finds that the other player plays the quantum game, and also finds that the other player finds that the player is considering the classical game. We shall show that the result of the game strictly depends on the sequence of viewpoints of the players.

To make the paper self-contained, we give the important preliminaries from theory of games with unawareness based on [20]. Then, we recall the idea of quantum duopoly introduced in [21].

## 2. Preliminaries

This section is based on the work in [20]. The work in [16] gives the reader comparable preliminaries on games with unawareness.

### 2.1. Normal form Games with Unawareness

Let $G=(N,{\left(S_{i}\right)}_{i\in N},{\left(u_{i}\right)}_{i\in N})$ be a normal-form game. This is the game played by the players and considered by the modeler. The concept of games with unawareness assumes that a player may not be aware of the full description of *G*. Therefore, $G_{v}=\left(N_{v},{\left({\left(S_{i}\right)}_{v}\right)}_{i\in N_{v}},{\left({\left(u_{i}\right)}_{v}\right)}_{i\in N_{v}}\right)$ denotes how player v’s views the game for v∈N. That is, the player v∈N views the set of players, the sets of players’ strategies, and the payoff functions as $N_{v}$, ${\left(S_{i}\right)}_{v}$ and ${\left(u_{i}\right)}_{v}$, respectively. In general, each player also considers how each of the other players views the game. Formally, given a finite sequence of players $v=(i_{1},\ldots ,i_{n})$, there is associated a game $G_{v}=\left(N_{v},{\left({\left(S_{i}\right)}_{v}\right)}_{i\in N_{v}},{\left({\left(u_{i}\right)}_{v}\right)}_{i\in N_{v}}\right)$. The game $G_{v}$ describes the situation in which player $i_{1}$ considers that player $i_{2}$ considers that …player $i_{n}$ is considering the game $G_{v}$. A sequence *v* is called a view. The empty sequence v=∅ is assumed to be the modeler’s view, i.e., $G_{\emptyset }=G$. We denote an action profile $\prod_{i\in N_{v}}s_{i}$ in $G_{v}$, where $s_{i}\in {\left(S_{i}\right)}_{v}$ by ${\left(s\right)}_{v}$. The concatenation of two views $\bar{v}=\left(i_{1},\ldots ,i_{n}\right)$ followed by $\tilde{v}=\left(j_{1},\ldots ,j_{n}\right)$ is defined as v=v¯^v˜=(i1,…,in,j1,…,jn). The set of all potential views is $V={⋃}_{n=0}^{\infty }N^{\left(n\right)}$ where $N^{\left(n\right)}=\prod_{j=1}^{n}N$ and $N^{\left(0\right)}=\emptyset$.

**Definition** **1.** *A collection ${\left\{G_{v}\right\}}_{v\in V}$ where V⊂V is a collection of finite sequences of players is called a normal-form game with unawareness (in a weak sense), and the collection of views V is called its set of relevant views if ${\left\{G_{v}\right\}}_{v\in V}$ and V satisfy the following conditions.*

*1*. *For every v∈V,*
  (1) v^v∈Vif and only ifv∈Nv.
*2*. *For every v^v˜∈V,*
  (2) v∈V,∅≠Nv^v˜⊂Nv,∅≠(Si)v^v˜⊂(Si)vfor alli∈Nv^v˜.
*3*. *If v^v^v¯∈V, then*
  (3) v^v^v^v¯∈VandGv^v^v¯=Gv^v^v^v¯.

The first property indicates what views are relevant. If the set of players $N_{1}$ seen by player 1 does not contain player 3, i.e., $3\notin N_{1}$, the view 1^3 what player 1 thinks that player 3 is considering is not relevant for player 1. Therefore, 1^3∉V.

The second property states that if player 1 thinks that player 2 is considering a player or a strategy as a part of the game, he/she takes those elements into account in the game considered by himself.

The third property say that if player 1 finds a game $G_{1}$, he/she also finds that he/she has that perception, i.e., G1^1=G1.

Games of the form $G_{v}$ that correspond to some views and the game $G_{\emptyset }$ of the modeler may differ in the number of players. As the payoffs results from strategies chosen by all the players, the payoffs in $G_{v}$ may not be uniquely determined. The fourth property indicates that the payoffs in the restricted game are the payoffs in the game with more players by adding some strategy profiles of these players. In other words, a restricted game cannot imply new payoffs.

### 2.2. Extended Nash Equilibrium

A Nash equilibrium [22] is a basic solution concept in a normal-form game.

**Definition** **2.** A strategy profile $s^{*}=\left(s_{1},s_{2},\ldots ,s_{n}\right)$ is a Nash equilibrium if for each player i∈{1,…,n} and each strategy $s_{i}$ of player i

(4) $$u_{i}\left(s^{*}\right)\geq u_{i}\left(s_{i},s_{-i}^{*}\right),$$

where $s_{-i}^{*}:={\left(s_{j}\right)}_{j\neq i}$.

The first step in defining the notion of Nash-type equilibrium for a normal-form game with unawareness is to redefine the notion of strategy profile.

**Definition** **3.** *Let ${\left\{G_{v}\right\}}_{v\in V}$ be a normal-form game with unawareness. An extended strategy profile (ESP) in ${\left\{G_{v}\right\}}_{v\in V}$ is a collection of strategy combinations ${\left\{{\left(\sigma \right)}_{v}\right\}}_{v\in V}$, where ${\left(\sigma \right)}_{v}$ is a strategy profile in the game $G_{v}$ such that for every v^v^v¯∈V holds*

(5) (σv)v=(σv)v^vas well as(σ)v^v^v¯=(σ)v^v^v^v¯.

As an illustration of Equation (5), let us consider the game $G_{12}$—the game that player 1 finds that player 2 is considering. If player 1 thinks that player 2 chooses strategy ${\left(\sigma_{2}\right)}_{12}$ in $G_{12}$, he/she must assume the same strategy in $G_{1}$, which is the game that he/she considers, i.e., ${\left(\sigma_{2}\right)}_{1}={\left(\sigma_{2}\right)}_{12}$.

The next step is an extension of rationalizability from normal-form games to the games with unawareness.

**Definition** **4.** *An extended strategy profile ${\left\{{\left(\sigma \right)}_{v}\right\}}_{v\in V}$ in a game with unawareness is called extended rationalizable if for every v^v∈V strategy ${\left(\sigma_{v}\right)}_{v}$ is a best reply to (σ−v)v^v in Gv^v.*

Let us consider a normal-form game with unawareness ${\left\{G_{v}\right\}}_{v\in V}$. Given a relevant view v∈V, the views as seen from *v* are defined to be Vv={v˜∈V:v^v˜∈V}. Then, the game with unawareness as seen from *v* is defined by {Gv^v˜}v˜∈Vv.

**Definition** **5.** *An extended strategy profile ${\left\{{\left(\sigma \right)}_{v}\right\}}_{v\in V}$ in a game with unawareness is called an extended Nash equilibrium (ENE) if it is rationalizable and for all $v,\bar{v}\in V$, such that*

(6) {Gv^v˜}v˜∈Vv={Gv¯^v˜}v˜∈Vv¯

*the following is satisfied; ${\left(\sigma \right)}_{v}={\left(\sigma \right)}_{\bar{v}}$.*

Definition 5 requires that each strategy of the profile is a best reply to the other strategies of that profile. According to Definition 4, the strategy ${\left(\sigma_{2}\right)}_{1}$ of player 2 in the game of player 1 is a best reply to player 1’s strategy ${\left(\sigma_{1}\right)}_{12}$ in the game $G_{12}$. Moreover, ${\left(\sigma_{1}\right)}_{12}$ is a best reply to strategy ${\left(\sigma_{2}\right)}_{121}$.

The following proposition [20] proves that ENE coincides with the standard Nash equilibrium for normal-form games if all views share the same perception of the game. Therefore, it is useful for determining extended Nash equilibria.

**Proposition** **1.** *Let G be a normal-form game and let ${\left\{G_{v}\right\}}_{v\in V}$ be a normal-form game with unawareness such that, for some v∈V, the equation Gv^v¯=G holds for every $\bar{v}$ such that v^v¯∈V. Let σ be a strategy profile in G. Then,*

*1*. *σ is rationalizable for G if and only if ${\left(\sigma \right)}_{v}=\sigma$ is part of an extended rationalizable profile in ${\left\{G_{v}\right\}}_{v\in V}$.*
*2*. *σ is a Nash equilibrium for G if and only if ${\left(\sigma \right)}_{v}=\sigma$ is part of on an extended Nash equilibrium for ${\left\{G_{v}\right\}}_{v\in V}$ and this ENE satisfies (σ)v=(σ)v^v¯.*

**Remark** **1.** *We see from Equations (3) and (5) that, for every v^v^v¯∈V, a normal-form game Gv^v^v¯ and a strategy profile (σ)v^v^v¯ determine the games and profiles in the form Gv^v^…^v^v¯ and (σ)v^v^…^v^v¯, respectively, for example, $G_{121}$ determines $G_{122\ldots 21}$. Therefore, in general, a game with unawareness ${\left\{G_{v}\right\}}_{v\in V}$ and an extended strategy profile ${\left\{{\left(\sigma \right)}_{v}\right\}}_{v\in V}$ are defined by ${\left\{G_{v}\right\}}_{v\in N\cup \{\emptyset \}}$ and ${\left\{{\left(\sigma \right)}_{v}\right\}}_{v\in N\cup \{\emptyset \}}$, respectively, where*

(7) $$N=\{v\in V|v=\left(i_{1},\ldots ,i_{n}\right)\;with\;i_{k}\neq i_{k+1}\;for\;all\;k\}.$$

*Then, we get ${\left\{G_{v}\right\}}_{v\in V}$ from ${\left\{G_{v}\right\}}_{v\in N\cup \{\emptyset \}}$ by setting $G_{\tilde{v}}=G_{v}$ for $v=(i_{1},\ldots ,i_{n})\in N$ and*

(8) $$\tilde{v}\;=\;\left(i_{1},\ldots ,i_{k},i_{k},i_{k+1},\ldots ,i_{n}\right)\in V.$$

## 3. Quantum Cournot’s Duopoly

The Li–Du–Massar (LDM) protocol [21] is a quantum scheme for duopoly problems. It can be treated as a minimal quantum model of a two-player strategic-form game of continuum of strategies. The LDM model creates a correlation of players’ strategies that enables the players to reach an optimal Nash equilibrium result. It is not possible when the players play Cournot’s duopoly in the classical way.

### 3.1. Classical Case

**Cournot’s duopoly** is one of the earliest economic models of competition between two players [23]. Each player offers a quantity of a homogeneous product which affects the price of the product and his gain. The price of the product is a decreasing function that depends on the total quantity. Formally, the Cournot duopoly can be seen as a strategic form game $\left(N,{\left(S_{i}\right)}_{i\in N},{\left(u_{i}\right)}_{i\in N}\right)$ with the components defined as follows:

1. the set of players is N={1,2},
2. the strategy set of player *i* is $S_{i}=\left[0,\infty \right)$,
3. player *i*’s payoff function $u_{i}$ is given by formula
  (9) $$u_{i}\left(q_{1},q_{2}\right)=q_{i}P\left(q_{1},q_{2}\right)-cq_{i},\;q_{1},q_{2}\in \left[0,\infty \right),$$
  where $P(q_{1},q_{2})$ represents the price of the product,
  (10) $$P\left(q_{1},q_{2}\right)=\left\{\begin{array}{cc} a-q_{1}-q_{2} & \mathrm{if}\;q_{1}+q_{2}<a,\\ 0 & \mathrm{if}\;q_{1}+q_{2}\geq a, \end{array}\right.$$
  and a marginal cost *c* satisfies a>c>0.

The game so defined has exactly one Nash equilibrium $\left(q_{1}^{*},q_{2}^{*}\right)=\left(\left(a-c\right)/3,\left(a-c\right)/3\right)$ with the payoff equal to ${\left(a-c\right)}^{2}/9$ for each player. One can check that the Nash equilibrium in the Cournot competition is not Pareto optimal. The players can benefit from playing strategy profile $\left(q_{1},q_{2}\right)=\left(\left(a-c\right)/4,\left(a-c\right)/4\right))$ and get ${\left(a-c\right)}^{2}/8$.

### 3.2. Quantum Case

The Li–Du–Massar quantum approach to the Cournot duopoly [21] (see [19] for more details) proceeds as follows. Let |00〉 be the initial state and $J\left(\gamma \right)=e^{-\gamma (a_{1}^{†}a_{2}^{†}-a_{1}a_{2})}$ be a unitary operator. The parameters γ≥0 and $a_{i}^{†}$ ($a_{i}$) represent the creation (annihilation) operator of electromagnetic field *i*. The player *i*’s strategies are unitary operators of the form

(11) $$D_{i}\left(x_{i}\right)=e^{x_{i}\left(a_{i}^{†}-a_{i}\right)/\sqrt{2}},x_{i}\in \left[0,\infty \right),i=1,2.$$

Then, the operator J(γ) and the strategy profile $D_{1}\left(x_{1}\right)⊗D_{2}\left(x_{2}\right)$ determine the final state $|\Psi_{f}\rangle$,

(12) $$|\Psi_{f}\rangle =J^{†}\left(\gamma \right)\left(D_{1}\left(x_{1}\right)⊗D_{2}\left(x_{2}\right)\right)J\left(\gamma \right)|00\rangle .$$

The quantity $q_{i}$ (in the case of Bertrand duopoly it is the price $p_{i}$) is then obtained by acting the measurement operator $X_{i}=\left(a_{i}^{†}+a_{i}\right)/\sqrt{2}$ on the state $|\Psi_{f}\rangle$. The result is

(13) $$\begin{array}{c} \langle \Psi_{f}|X_{1}|\Psi_{f}\rangle =x_{1}\cosh\gamma +x_{2}\sinh\gamma ,\\ \langle \Psi_{f}|X_{2}|\Psi_{f}\rangle =x_{2}\cosh\gamma +x_{1}\sinh\gamma . \end{array}$$

After normalization of (13), done by setting

(14) $$x_{i}\mapsto D\left(\frac{x_{i}}{e^{\gamma }}\right),$$

the resulting quantities become

(15) $$q_{1}=\frac{x_{1}\cosh\gamma +x_{2}\sinh\gamma }{e^{\gamma }},\;q_{2}=\frac{x_{2}\cosh\gamma +x_{1}\sinh\gamma }{e^{\gamma }}.$$

We get the quantum approach to the classical Cournot duopoly by substituting Equation (15) into Equation (9),

(16) $$u_{1(2)}\left(x_{1},x_{2},\gamma \right)=\left\{\begin{array}{cc} q_{1(2)}\left(a-c-\left(x_{1}+x_{2}\right)\right) & \mathrm{if}\;(x_{1}+x_{2})<a,\\ -cq_{1(2)} & \mathrm{if}\;(x_{1}+x_{2})\geq a. \end{array}\right.$$

From Equation (11), the strategies of player *i* are identified with choosing $x_{i}\in \left[0,\infty \right)$. Furthermore, Equation (15) shows that the scheme correlates the players’ strategies and the higher the value of γ, the stronger correlation between $x_{1}$ and $x_{2}$.

## 4. Quantum Cournot Duopoly with Unawareness

**Figure 1:**
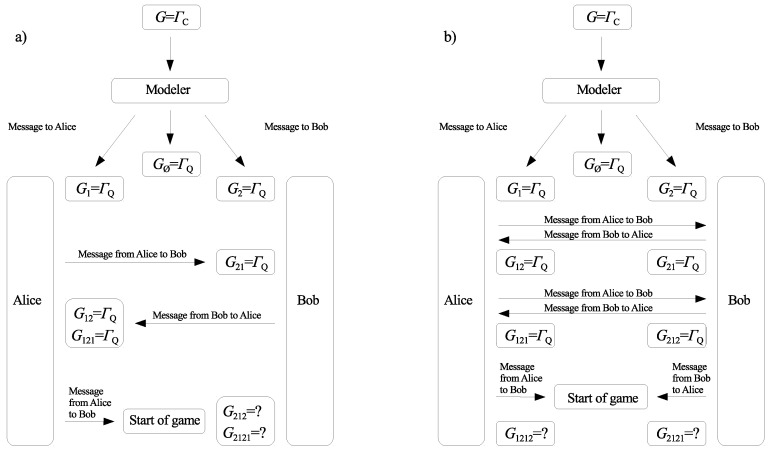
Two examples of exchanging messages by the players: (**a**) messages sent sequentially, (**b**) messages sent simultaneously.

In [16,17], we pointed out that the concept of games with unawareness can be useful when the fact of playing a quantum game is not common knowledge among the players. This may be the case when the players are far away from each other, and a third party is obliged to prepare the game (classical or quantum). After the third party prepares the quantum game, he/she sends the message to the players to inform them that they play the quantum game, and not the classical one. When the players receive the message, player i∈{1,2} perceives the game as being quantum, i.e., $G_{i}=\Gamma_{Q}$. However, this fact is not common knowledge among players 1 and 2. Player 2 finds that player 1 is considering the quantum game, i.e., $G_{21}=\Gamma_{Q}$, if player 1 confirms he/she received the message from the third party. Similarly, player 1 receiving a message from player 2 will learn that player 2 is considering the quantum game, $G_{12}=\Gamma_{Q}$. Two examples of possible scenarios for exchanging players’ messages are shown in Figure 1. The two methods determine games with unawareness that are described by collections of games $\left\{G_{v}^{a}\right\}$ and $\left\{G_{v}^{b}\right\}$,

(17) $$G_{v}^{a}=\left\{\begin{array}{cc} \Gamma_{Q} & \mathrm{if}\;v\in \{1,2,12,21,121\}\\ \Gamma_{C} & \mathrm{otherwise}, \end{array}\right.\;G_{v}^{b}=\left\{\begin{array}{cc} \Gamma_{Q} & \mathrm{if}\;v\in \{1,2,12,21,121,212\}\\ \Gamma_{C} & \mathrm{otherwise}. \end{array}\right.$$

In what follows, we show that the order in which the players send messages to each other has a significant impact on the rational result of the game. It can be seen by comparing extended Nash equilibria in the games $\left\{G_{v}^{a}\right\}$ and $\left\{G_{v}^{b}\right\}$.

Recall that $\left(\sigma_{1}^{c},\sigma_{2}^{c}\right)=\left(\left(a-c\right)/3,\left(a-c\right)/3\right)$ is the unique Nash equilibrium in the classical Cournot duopoly. By Proposition 1, the strategy profile ((a−c)/3,(a−c)/3) is part of an ENE for

(18) v∈{1212,12121,212,2121,…}.

This means that

(19) $${\left(\sigma \right)}_{1212}={\left(\sigma \right)}_{12121}={\left(\sigma \right)}_{212}={\left(\sigma \right)}_{2121}={\left(\sigma \right)}_{21212}=\left(\frac{a-c}{3},\frac{a-c}{3}\right).$$

Let us now determine the strategy profile ${\left(\sigma \right)}_{121}$. By Definition 3,

(20) $${\left(\sigma_{2}\right)}_{121}={\left(\sigma_{2}\right)}_{1212}=\frac{a-c}{3}.$$

According to Definition 4, Alice’s strategy ${\left(\sigma_{1}\right)}_{121}$ is a best reply to ${\left(\sigma_{2}\right)}_{121}=\left(a-c\right)/3$ in the game $G_{121}=\Gamma_{Q}$. Substituting (a−c)/3 into Equation (16), we deduce that

(21) $${\left(\sigma_{1}\right)}_{121}=\frac{1}{6}\left(a-c\right)\left(2-\tanh\gamma \right).$$

As a result,

(22) $${\left(\sigma \right)}_{121}=\left(\frac{1}{6}\left(a-c\right)\left(2-\tanh\gamma \right),\frac{a-c}{3}\right).$$

Similarly,

(23) $${\left(\sigma_{1}\right)}_{12}={\left(\sigma_{1}\right)}_{121}=\frac{1}{6}\left(a-c\right)\left(2-\tanh\gamma \right).$$

As ${\left(\sigma_{2}\right)}_{12}$ is a best reply to ${\left(\sigma_{1}\right)}_{12}={\left(\sigma_{1}\right)}_{121}=\left(1/6\right)k\left(2-\tanh\gamma \right)$, we conclude that

(24) $${\left(\sigma_{2}\right)}_{12}=\frac{1}{12}\left(a-c\right)\left(4-\tanh\gamma +{\left(\tanh\gamma \right)}^{2}\right).$$

In the case of ${\left(\sigma \right)}_{1}={\left(\sigma_{1},\sigma_{2}\right)}_{1}$, we have ${\left(\sigma_{2}\right)}_{1}={\left(\sigma_{2}\right)}_{12}$ given by Equation (24). Now, player 1’s best reply to ${\left(\sigma_{2}\right)}_{12}$ in the game $G_{1}=\Gamma_{Q}$ is

(25) $${\left(\sigma_{1}\right)}_{1}=\frac{1}{24}\left(a-c\right)\left(8-3\tanh\gamma -{\left(\tanh\gamma \right)}^{3}\right).$$

In the same manner, we can see that the strategy profile ${\left(\sigma \right)}_{2}$ seen by player 2 is given by

(26) $${\left(\sigma \right)}_{2}=\left(\frac{1}{6}\left(a-c\right)\left(2-\tanh\gamma \right),\frac{1}{12}\left(a-c\right)\left(4-\tanh\gamma +{\left(\tanh\gamma \right)}^{2}\right)\right).$$

The strategy profile that is actually played by the players corresponds to ${\left(\sigma \right)}_{\emptyset }={\left(\sigma_{1},\sigma_{2}\right)}_{\emptyset }$. As ${\left(\sigma_{1}\right)}_{\emptyset }={\left(\sigma_{1}\right)}_{1}$ and ${\left(\sigma_{2}\right)}_{\emptyset }={\left(\sigma_{2}\right)}_{2}$, we conclude that

(27) $$\begin{array}{c} {\left(\sigma \right)}_{\emptyset }={\left(\sigma \right)}_{1}\\ \;=\left(\frac{1}{24}\left(a-c\right)\left(8-3\tanh\gamma -{\left(\tanh\gamma \right)}^{3}\right),\frac{1}{12}\left(a-c\right)\left(4-\tanh\gamma +{\left(\tanh\gamma \right)}^{2}\right)\right). \end{array}$$

The result ${\left(\sigma \right)}_{\emptyset }$ of $\left\{G_{v}^{a}\right\}$ implies

(28) $$\begin{array}{cc} u_{1}\left({\left(\sigma \right)}_{\emptyset },\gamma \right) & =\frac{e^{2\gamma }{\left(6+7e^{2\gamma }+3e^{4\gamma }\right)}^{2}{\left(a-c\right)}^{2}}{72{\left(1+e^{2\gamma }\right)}^{5}}\underset{\gamma \to \infty }{\to }\frac{{\left(a-c\right)}^{2}}{8}, \end{array}$$

(29) $$\begin{array}{cc} u_{2}\left({\left(\sigma \right)}_{\emptyset },\gamma \right) & =\frac{6+11e^{2\gamma }+12e^{4\gamma }+3e^{6\gamma }}{6+13e^{2\gamma }+10e^{4\gamma }+3e^{6\gamma }}\cdot u_{1}\left({\left(\sigma \right)}_{\emptyset },\gamma \right)\underset{\gamma \to \infty }{\to }\frac{{\left(a-c\right)}^{2}}{8}. \end{array}$$

The analysis, which is similar to that of $\left\{G_{v}^{a}\right\}$, shows that the result of playing an extended Nash equilibrium in $\left\{G_{v}^{b}\right\}$ is

(30) $${\left(\sigma \right)}_{\emptyset }=\left(\frac{1}{24}\left(a-c\right)\left(8-3\tanh\gamma -{\left(\tanh\gamma \right)}^{3}\right),\frac{1}{24}\left(a-c\right)\left(8-3\tanh\gamma -{\left(\tanh\gamma \right)}^{3}\right)\right).$$

Then profile (30) implies the payoffs

(31) $$\begin{array}{cc} \; & u_{1}\left({\left(\sigma \right)}_{\emptyset },\gamma \right)=u_{2}\left({\left(\sigma \right)}_{\emptyset },\gamma \right) \end{array}$$

(32) $$\begin{array}{c} =\frac{e^{2\gamma }\left(3+3e^{2\gamma }+2e^{4\gamma }\right)\left(3+6e^{2\gamma }+6e^{4\gamma }+e^{6\gamma }\right){\left(a-c\right)}^{2}}{18{\left(1+e^{2\gamma }\right)}^{6}}\underset{\gamma \to \infty }{\to }\frac{{\left(a-c\right)}^{2}}{9}. \end{array}$$

## 5. General Framework

The way of finding an extended Nash equilibria in $\left\{G_{v}^{a}\right\}$ and $\left\{G_{v}^{b}\right\}$, given by Equation (17), can be generalized to any two-person game with unawareness in which higher-order iteration of the awareness of players 1 and 2 is associated with the same games.

**Proposition** **2.** *Let ${\left\{G_{v}\right\}}_{v\in V}$ be a two-person game with unawareness and ${\mathrm{br}}_{i}^{v}\left(\cdot \right)$ be a best reply correspondence of player i in the game $G_{v}$. Let A and B be normal-form games, such that for some $\bar{v}\in V_{1}=\left\{1,12,121,\ldots \right\}$ and $\tilde{v}\in V_{2}=\left\{2,21,212,\ldots \right\}$ we have Gv¯^v=A and Gv˜^v=B for every v¯^v∈V1, v˜^v∈V2.*

*A strategy profile ${\left(\sigma_{1},\sigma_{2}\right)}_{\emptyset }$ in an extended Nash equilibrium ${\left\{\left(\sigma_{1},\sigma_{2}\right)\right\}}_{v\in V}$ of ${\left\{G_{v}\right\}}_{v\in V}$ satisfies*

(33) $$\begin{array}{cc} \; & {\left(\sigma_{1}\right)}_{\emptyset }\in {\mathrm{br}}_{1}^{1}\circ {\mathrm{br}}_{2}^{12}\circ {\mathrm{br}}_{1}^{121}\circ \ldots \circ {\mathrm{br}}_{i}^{\bar{v}}\left(\sigma_{-i}^{A}\right) \end{array}$$

(34) $$\begin{array}{cc} \; & {\left(\sigma_{2}\right)}_{\emptyset }\in {\mathrm{br}}_{2}^{2}\circ {\mathrm{br}}_{1}^{21}\circ {\mathrm{br}}_{2}^{212}\circ \ldots \circ {\mathrm{br}}_{j}^{\tilde{v}}\left(\sigma_{-j}^{B}\right), \end{array}$$

*for some Nash equilibria $\left(\sigma_{1}^{A},\sigma_{2}^{A}\right)$ and $\left(\sigma_{1}^{B},\sigma_{2}^{B}\right)$ of A and B, respectively (provided that the Nash equilibria of A and B exist).*

**Proof.**  By Proposition 1, Nash equilibrium strategies for *A* and *B* are parts of ENE starting from views v¯^v∈V1 and v˜^v∈V2, respectively. By the definition of ENE, ${\left(\sigma_{1}\right)}_{\emptyset }={\left(\sigma_{1}\right)}_{1}$ is a best reply to ${\left(\sigma_{2}\right)}_{1}={\left(\sigma_{2}\right)}_{12}$. If (σ2)12^v=σ2A, then

(35) $${\left(\sigma_{1}\right)}_{\emptyset }\in {\mathrm{br}}_{1}^{1}\left(\sigma_{2}^{A}\right),$$

which ends the proof. Otherwise, ${\left(\sigma_{2}\right)}_{12}$ is a best reply to ${\left(\sigma_{1}\right)}_{12}$. Now, if (σ1)12^v=σ1A, then ${\left(\sigma_{2}\right)}_{12}\in {\mathrm{br}}_{2}^{12}\left(\sigma_{1}^{A}\right)$, and therefore

(36) $${\left(\sigma_{1}\right)}_{\emptyset }\in {\mathrm{br}}_{1}^{1}\left({\mathrm{br}}_{2}^{12}\left(\sigma_{1}^{A}\right)\right).$$

Continuing in this way, we arrive at the conclusion that ${\left(\sigma_{i}\right)}_{\bar{v}}$ is a best reply to (σ−i)v¯^i. By assumption, (σ−i)v¯^i=σ−iA. As a result, ${\left(\sigma_{i}\right)}_{\bar{v}}\in {\mathrm{br}}_{i}^{\bar{v}}\left(\sigma_{-i}^{A}\right)$, and, together with the previous steps,

(37) (σ1)∅∈br11(br212(…(briv¯((σ−i)v¯^i))).

 □

An immediate consequence of Proposition 2 is an explicit formula for computing the result of an ENE in a wide class of the Cournot duopoly with unawareness.

**Proposition** **3.** *Let $\Gamma_{Q}$ be the quantum Cournot duopoly and $\Gamma_{C}$ be its classical counterpart (γ=0). Let ${\left\{G_{v}\right\}}_{v\in V}$ be a game with unawareness, where*

(38) $$G_{v}=\left\{\begin{array}{cc} \Gamma_{Q} & \mathrm{if}\;v\in \left\{1,12,\ldots ,\bar{v}\right\}\cup \left\{2,21,\ldots ,\tilde{v}\right\},\\ \Gamma_{C} & otherwise, \end{array}\right.$$

*and $\bar{v}\in V_{1}$, $\tilde{v}\in V_{2}$.*

*The strategy profile ${\left(\sigma_{1},\sigma_{2}\right)}_{\emptyset }$ in an extended Nash equilibrium ${\left\{\left(\sigma_{1},\sigma_{2}\right)\right\}}_{v\in V}$ of ${\left\{G_{v}\right\}}_{v\in V}$ and is of the form*

(39) $${\left(\sigma_{1},\sigma_{2}\right)}_{\emptyset }=\left(x_{1}^{n},x_{2}^{m}\right),$$

*where*

(40) $$x_{i}^{y}=\frac{\left(a-c\right)\left(3+{\left(-\frac{1}{2}\right)}^{y}\tanh\gamma {\left(1+\tanh\gamma \right)}^{y}\right)}{3(3+\tanh\gamma )}$$

*and n and m are the lengths of the sequences $\bar{v}$ and $\tilde{v}$, respectively.*

**Proof.**  We prove the result for player 1. The proof is conducted by induction on the length of $\bar{v}\in V_{1}$. First, we prove that Equation (40) holds for $|\bar{v}|=0$. Then, ${\left\{G_{v}\right\}}_{v\in V_{1}}=\left\{\Gamma_{C}\right\}$ and $\left(x_{1}^{0},x_{2}^{0}\right)=\left(\left(a-c\right)/3,\left(a-c\right)/3\right)$. It follows from Proposition 1 the result ${\left(\sigma_{1},\sigma_{2}\right)}_{\emptyset }$ predicted by an ENE in $\left\{\Gamma_{C}\right\}$ is a Nash equilibrium in $\Gamma_{C}$. The Cournot duopoly game has the unique Nash equilibrium ((a−c)/3,(a−c)/3) (see, for example, [24]). As a result, Formula (40) is true for y=0.

Assume by induction that Equation (40) holds for *n*. We will prove that it holds for n+1. Let us consider ${\left\{G_{v}\right\}}_{v\in V_{1}}$ with $|\bar{v}|=n+1$. As $G_{v}=\Gamma_{Q}$ for $v\in \{1,12,\ldots ,\bar{v}\}$, it follows from Proposition 2 that

(41) $${\left(\sigma_{1}\right)}_{\emptyset }={\left(\sigma_{1}\right)}_{1}={\mathrm{br}}_{1}^{1}\circ {\mathrm{br}}_{2}^{12}\circ \ldots \circ {\mathrm{br}}_{i}^{\bar{v}}\left(\frac{a-c}{3}\right).$$

The best reply correspondence associated with $\Gamma_{Q}$ is a function ${\mathrm{br}}_{i}:\left[0,\infty \right)\to \left[0,\infty \right)$,

(42) $${\mathrm{br}}_{i}^{v}\left(x\right)=\frac{1}{2}\left(a-c-x-x\tanh\gamma \right),\;v\in \left\{1,2,\ldots ,\bar{v}\right\}.$$

Note that ${\left(\sigma_{1}\right)}_{\emptyset }={\mathrm{br}}_{1}^{1}\left(\xi \right)$, where $\xi ={\mathrm{br}}_{2}^{12}\circ \ldots \circ {\mathrm{br}}_{i}^{\bar{v}}\left(\left(a-c\right)/3\right)$. By the induction hypothesis, ξ is given by the right-hand side of Equation (40). As a result,

$$\begin{array}{cc} {\left(\sigma_{1}\right)}_{\emptyset } & ={\mathrm{br}}_{1}\left(\frac{\left(a-c\right)\left(3+{\left(-\frac{1}{2}\right)}^{n}\tanh\gamma {\left(1+\tanh\gamma \right)}^{n}\right)}{3(3+\tanh\gamma )}\right) \end{array}$$

(43) $$\begin{array}{cc} \; & =\frac{1}{2}\left(a-c-\frac{\left(a-c\right)\left(3+{\left(-\frac{1}{2}\right)}^{n}\tanh\gamma {\left(1+\tanh\gamma \right)}^{n}\right)}{3(3+\tanh\gamma )}\left(1+\tanh\gamma \right)\right) \end{array}$$

(44) $$\begin{array}{cc} \; & =\frac{\left(a-c\right)\left(3+{\left(-\frac{1}{2}\right)}^{n+1}\tanh\gamma {\left(1+\tanh\gamma \right)}^{n+1}\right)}{3(3+\tanh\gamma )}, \end{array}$$

which is what we needed to show. □

**Remark** **2.** *Note that for γ=0, each element of the collection $\left\{G_{v}\right\}$ given by Equation (38) is the classical Cournot duopoly game (in other words, playing the classical game is common knowledge among the players). The strategy $x_{i}^{y}$ takes into account that case, i.e., $x_{i}^{y}$ is equal to the classical Nash equilibrium strategy (a−c)/3 for γ=0. Note also that $x_{i}^{y}\leq \left(a-c\right)/3$ for every γ∈[0,∞). This means that the players playing according to $\left(x_{i}^{m},x_{i}^{n}\right)$.*

As an application of Proposition 3, we reconsider the example given by Equation (17).

**Example** **1.** *Let us consider $\left\{G_{v}^{a}\right\}$ and $\left\{G_{v}^{b}\right\}$ given by Equation (17). Then, in terms of Equation (38), $\bar{v}=121,\tilde{v}=21$ in the case of $\left\{G_{v}^{a}\right\}$ and $\bar{v}=121,\tilde{v}=212$ in $\left\{G_{v}^{b}\right\}$. According to Lemma 3, the actual strategy profile played in games $\left\{G_{v}^{a}\right\}$ and $\left\{G_{v}^{b}\right\}$ is ${\left(\sigma_{1},\sigma_{2}\right)}_{\emptyset }=\left(x_{1}^{3},x_{2}^{2}\right)$ and ${\left(\sigma_{1},\sigma_{2}\right)}_{\emptyset }=\left(x_{1}^{3},x_{2}^{3}\right)$, respectively, where*

(45) $$x_{i}^{3}=\frac{1}{24}\left(a-c\right)\left(8-3\tanh\gamma -{\left(\tanh\gamma \right)}^{3}\right),\;x_{i}^{2}=\frac{1}{12}\left(a-c\right)\left(4-\tanh\gamma +{\left(\tanh\gamma \right)}^{2}\right).$$

**Figure 2:**
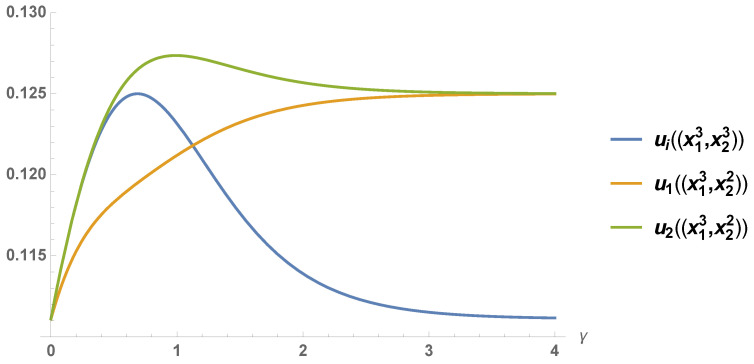
The extended Nash equilibrium (ENE) payoffs (for a−c=1) associated with the profiles $\left(x_{1}^{3},x_{2}^{3}\right)$ and $\left(x_{1}^{3},x_{2}^{2}\right)$ depending on the value of γ.

*As shown in Figure 2, the result of the game varies depending on the strategy profile $\left(x_{1}^{3},x_{2}^{2}\right)$ and $\left(x_{1}^{3},x_{2}^{3}\right)$. In the first case, the resulting payoff converges to the Pareto optimal outcome ${\left(a-c\right)}^{2}/8$, as γ increases to infinity. The second case implies the equilibrium outcome goes to ${\left(a-c\right)}^{2}/9$, as γ goes to infinity.*

## 6. Bertrand Price Competition

The Bertrand model [25] was proposed as an alternative to the Cournot model [23]. In the Bertrand model of competition, two players compete in the price of a homogeneous product. The firm with a lower price captures the entire market. If both firms charge the same price, they split the market equally. To be more specific, it is assumed that the payoff function $u_{i}$ of player i∈{1,2} is a function of prices $p_{1}$ and $p_{2}$ determined by player 1 and 2, respectively. Moreover, we assume that each firm has the same marginal cost *c* such that 0≤c<a. Then, the payoff function of player 1 is

(46) $$u_{1}\left(p_{1},p_{2}\right)=\left\{\begin{array}{cc} \left(p_{1}-c\right)\left(a-p_{1}\right) & \mathrm{if}\;p_{1}<p_{2}\;\mathrm{and}\;p_{1}\leq a,\\ \frac{1}{2}\left(p_{1}-c\right)\left(a-p_{1}\right) & \mathrm{if}\;p_{1}=p_{2}\;\mathrm{and}\;p_{1}\leq a,\\ 0 & \mathrm{otherwise}. \end{array}\right.$$

Similarly, the payoff function of player 2 is

(47) $$u_{2}\left(p_{1},p_{2}\right)=\left\{\begin{array}{cc} \left(p_{2}-c\right)\left(a-p_{2}\right) & \mathrm{if}\;p_{2}<p_{1}\;\mathrm{and}\;p_{2}\leq a,\\ \frac{1}{2}\left(p_{2}-c\right)\left(a-p_{2}\right) & \mathrm{if}\;p_{1}=p_{2}\;\mathrm{and}\;p_{2}\leq a,\\ 0 & \mathrm{otherwise}. \end{array}\right.$$

The game defined by Equations (46) and (47) has the unique Nash equilibrium $\left(p_{1}^{*},p_{2}^{*}\right)=\left(c,c\right)$ that arises from intersection of best reply functions $\beta_{1}\left(p_{2}\right)$ and $\beta_{2}\left(p_{1}\right)$,

(48) $$\begin{array}{c} \beta_{1}\left(p_{2}\right)=\left\{\begin{array}{cc} \left\{p_{1}|p_{1}>p_{2}\right\} & \mathrm{if}\;p_{2}<c,\\ \left\{p_{1}|p_{1}\geq c\right\} & \mathrm{if}\;p_{2}=c,\\ \emptyset & \mathrm{if}\;c<p_{2}\leq \frac{a+c}{2},\\ \left\{\frac{a+c}{2}\right\} & \mathrm{if}\;p_{2}>\frac{a+c}{2}, \end{array}\right.\\ \beta_{2}\left(p_{1}\right)=\left\{\begin{array}{cc} \left\{p_{2}|p_{2}>p_{1}\right\} & \mathrm{if}\;p_{1}<c,\\ \left\{p_{2}|p_{2}\geq c\right\} & \mathrm{if}\;p_{1}=c,\\ \emptyset & \mathrm{if}\;c<p_{1}\leq \frac{a+c}{2},\\ \left\{\frac{a+c}{2}\right\} & \mathrm{if}\;p_{1}>\frac{a+c}{2}. \end{array}\right. \end{array}$$

The equilibrium implies the payoff of 0 for both players.

According to the quantum model introduced in [21], the normalized players’ prices $p_{1}$ and $p_{2}$ are determined as functions $p_{i}:{\left[0,\infty \right)}^{3}\to \left[0,\infty \right)$ of $x_{1},x_{2}$ and a fixed entanglement parameter γ∈[0,∞),

(49) $$\left\{\begin{array}{c} p_{1}\left(x_{1},x_{2},\gamma \right)=\frac{x_{1}\cosh\gamma +x_{2}\sinh\gamma }{e^{\gamma }},\\ p_{2}\left(x_{1},x_{2},\gamma \right)=\frac{x_{2}\cosh\gamma +x_{1}\sinh\gamma }{e^{\gamma }}. \end{array}\right.$$

(50) $$\begin{array}{c} u_{1}^{Q}\left(x_{1},x_{2},\gamma \right)=\left\{\begin{array}{cc} \left(p_{1}\left(x_{1},x_{2},\gamma \right)-c\right)\left(a-p_{1}\left(x_{1},x_{2},\gamma \right)\right) & \mathrm{if}\;x_{1}<x_{2}\;\mathrm{and}\;p_{1}\left(x_{1},x_{2},\gamma \right)\leq a,\\ \frac{1}{2}\left(p_{1}\left(x_{1},x_{2},\gamma \right)-c\right)\left(a-p_{1}\left(x_{1},x_{2},\gamma \right)\right) & \mathrm{if}\;x_{1}=x_{2}\;\mathrm{and}\;x_{1}\leq a,\\ 0 & \mathrm{otherwise}, \end{array}\right. \end{array}$$

(51) $$\begin{array}{c} u_{2}^{Q}\left(x_{1},x_{2},\gamma \right)=\left\{\begin{array}{cc} \left(p_{2}\left(x_{1},x_{2},\gamma \right)-c\right)\left(a-p_{2}\left(x_{1},x_{2},\gamma \right)\right) & \mathrm{if}\;x_{2}<x_{1}\;\mathrm{and}\;p_{2}\left(x_{1},x_{2},\gamma \right)\leq a,\\ \frac{1}{2}\left(p_{2}\left(x_{1},x_{2},\gamma \right)-c\right)\left(a-p_{2}\left(x_{1},x_{2},\gamma \right)\right) & \mathrm{if}\;x_{1}=x_{2}\;\mathrm{and}\;x_{2}\leq a,\\ 0 & \mathrm{otherwise}. \end{array}\right. \end{array}$$

To determine extended Nash equilibria in a Bertrand duopoly example with unawareness, presented below, we need to find a player’s best reply to x=c. We describe the result in the form of the following lemma.

**Lemma** **1.** *Denote by $\Gamma_{C}^{B}$ and $\Gamma_{Q}^{B}$ the Bertrand duopoly examples given by Equations (46) and (47) and (50) and (51), respectively. The set of player i’s best reply to x=c is [c,∞) in the games $\Gamma_{C}^{B}$ and $\Gamma_{Q}^{B}$.*

**Proof.**  If $x_{2}<c$, then player 1 gets a negative payoff by choosing $x_{1}\leq x_{2}$. Indeed,

(52) $$p_{1}\left(x_{1},x_{2},\gamma \right)=\frac{x_{1}\cosh\gamma +x_{2}\sinh\gamma }{e^{\gamma }}-c<\frac{c\cosh\gamma +c\sinh\gamma }{e^{\gamma }}-c=0$$

and

(53) $$a-p_{1}\left(x_{1},x_{2},\gamma \right)=a-\frac{x_{1}\cosh\gamma +x_{2}\sinh\gamma }{e^{\gamma }}>a-c>0.$$

Therefore, according to Equation (50), it is optimal for player 1 to take $x_{1}>x_{2}$ and get the payoff of 0. Similarly, if $x_{2}=c$, then $x_{1}<x_{2}$ yields player 1 a negative payoff. For this reason, player 1’s best reply is $x_{1}\geq c$, for which he/she obtains 0. □

**Example** **2.** *Consider ${\left\{G_{v}\right\}}_{v\in V}$ with the components defined as follows,*

(54) $$G_{v}=\left\{\begin{array}{cc} \Gamma_{Q}^{B} & if\;v\in \{\emptyset ,1\},\\ \Gamma_{C}^{B} & otherwise. \end{array}\right.$$

*The collection ${\left\{G_{v}\right\}}_{v\in V}$ describes the case where player 1 is fully aware of playing the quantum game $\Gamma_{Q}^{B}$, whereas player 2 is completely unaware of playing $\Gamma_{Q}^{B}$. Moreover, player 1 finds that player 2 is considering the classical game $\Gamma_{C}^{B}$. Therefore, it is reasonable to think that player 1 is in a better strategic position than player 2.*

To find an extended Nash equilibrium, we first note that

(55) $$G_{2}=G_{12}=G_{21}=G_{121}=\ldots =\Gamma_{C}^{B}.$$

By Proposition 1, an extended Nash equilibrium satisfies

$${\left(\sigma \right)}_{2}={\left(\sigma \right)}_{12}={\left(\sigma \right)}_{21}={\left(\sigma \right)}_{121}=\ldots =\left(c,c\right).$$

According to Proposition 3, ${\left(\sigma_{1}\right)}_{\emptyset }={\left(\sigma_{1}\right)}_{1}={\mathrm{br}}_{1}\left(c\right)$. Therefore, by Lemma 1, ${\left(\sigma_{1}\right)}_{1}\in \left[c,\infty \right)$. To sum up, the result implied by a possible Nash equilibrium in the game given by Equation (54) is

(56) $${\left(\sigma_{1},\sigma_{2}\right)}_{\emptyset }\in \left\{\left(x_{1},c\right):x_{1}\geq c\right\}.$$

**Figure 3:**
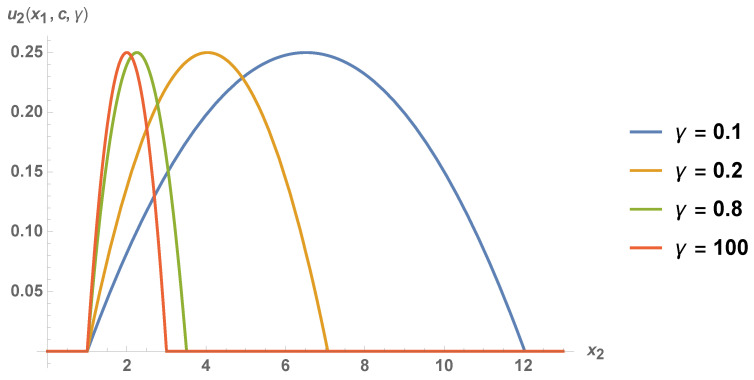
The payoff of player 2 corresponding to Equation (56) for fixed entanglement parameters γ and a−c=1.

The payoffs for players 1 and 2 corresponding to Equation (56) are illustrated in Figure 3, and they are given by the following formulas:

(57) $$\begin{array}{cc} u_{1}\left(x_{1},c,\gamma \right) & =0, \end{array}$$

(58) $$\begin{array}{cc} u_{2}\left(x_{1},c,\gamma \right) & =\left\{\begin{array}{cc} \frac{\left(x_{1}-c\right)\sinh\gamma \left(\left(a-c\right)\cosh\gamma +\left(a-x_{1}\right)\sinh\gamma \right)}{e^{-2\gamma }} & \mathrm{if}\;c\leq x_{1}\leq a+\left(a-c\right)\coth\gamma ,\\ 0 & \mathrm{otherwise}, \end{array}\right. \end{array}$$

where the form of piecewise function (58) follows from the fact that $p_{2}\left(x_{1},c,\gamma \right)\leq a$ if and only if $x_{1}\leq a+\left(a-c\right)\coth\gamma$. Thus, player 2 gets a positive payoff as long as $x_{1}\in \left(c,a+\left(a-c\right)\coth\gamma \right)$. In particular, he/she may obtain the monopoly payoff ${\left(a-c\right)}^{2}/4$. Indeed, from the first subfunction of (51) it may be concluded that

(59) $$p_{2}\left(x_{1},x_{2},\gamma \right)=\frac{a+c}{2}.$$

maximizes $u_{2}^{Q}\left(x_{1},x_{2},\gamma \right)$. From equation

(60) $$p_{2}\left(x_{1},c,\gamma \right)=\frac{c\cosh\gamma +x_{1}\sinh\gamma }{e^{\gamma }}=\frac{a+c}{2}$$

we obtain

(61) $$x_{1}=\frac{1}{2}\left(a+c+\left(a-c\right)\coth\gamma \right).$$

Therefore, if, in the equilibrium (56), player 1 chooses $x_{1}$ given by Equation (61), player 2 gets

(62) $$u_{2}\left(\frac{1}{2}\left(a+c+\left(a-c\right)\coth\gamma \right),c,\gamma \right)=\frac{1}{4}{\left(a-c\right)}^{2}.$$

## 7. Conclusions

Our research has shown that a rational result in the quantum duopolies depends on whether the players play the quantum game is common knowledge or not. The Pareto optimal outcome ${\left(a-c\right)}^{2}/8$ is achievable in the quantum Cournot duopoly with maximally correlated strategies if each player knows that he/she plays the quantum game, but he/she also has to know that the other player perceives the quantum game, and each player *i* finds that the other player finds that player *i* is considering the quantum game and so on. In case players’ perceptions are limited characteristics of the equilibrium payoff outcome varies depending on the level of awareness of the players. We have shown that an asymmetric distribution of players’ unawareness may be beneficial to the players in the quantum Cournot duopoly game, whereas rational strategies of equally unaware players imply the inefficient equilibrium outcome ${\left(a-c\right)}^{2}/9$.

The notion of game with unawareness finds also application in the quantum Bertrand duopoly. The example used in the paper indicates that the equilibrium result is more unified in the game with unawareness than in the case in which playing the quantum Bertrand duopoly is common knowledge.
